# Phasor map analysis to investigate Hutchinson–Gilford progeria cell under polarization-resolved optical scanning microscopy

**DOI:** 10.1038/s41598-022-05755-1

**Published:** 2022-01-31

**Authors:** Ali Mohebi, Aymeric Le Gratiet, Alberta Trianni, Fabio Callegari, Paolo Bianchini, Alberto Diaspro

**Affiliations:** 1 Nanoscopy and NIC@IIT, Italian Institute of Technology (IIT), Via Enrico Melen 83, 16152 Genova, Italy; 2 Department of Physics, University of Genova, Via Dodecaneso, 16146 Genova, Italy; 3 CNRS, Institut FOTON-UMR 6082, Université de Rennes 1, 22305 Lannion, France

**Keywords:** Biophysics, Optical physics, Microscopy, Multiphoton microscopy, Polarization microscopy

## Abstract

Polarized light scanning microscopy is a non-invasive and contrast-enhancing technique to investigate anisotropic specimens and chiral organizations. However, such arrangements suffer from insensitivity to confined blend of structures at sub-diffraction level. Here for the first time, we present that the pixel-by-pixel polarization modulation converted to an image phasor approach issues an insightful view of cells to distinguish anomalous subcellular organizations. To this target, we propose an innovative robust way for identifying changes in the chromatin compaction and distortion of nucleus morphology induced by the activation of the lamin-A gene from Hutchinson–Gilford progeria syndrome that induces a strong polarization response. The phasor mapping is evaluated based on the modulation and phase image acquired from a scanning microscope compared to a confocal fluorescence modality of normal cell opposed to the progeria. The method is validated by characterizing polarization response of starch crystalline granules. Additionally, we show that the conversion of the polarization-resolved images into the phasor could further utilized for segmenting specific structures presenting various optical properties under the polarized light. In summary, image phasor analysis offers a distinctly sensitive fast and easy representation of the polarimetric contrast that can pave the way for remote diagnosis of pathological tissues in real-time.

## Introduction

The perception of chromatin compartments as chiral-group structures composed of DNA double helix and proteins inside eukaryotic nuclei is crucial to identify enriched expressed genes regions based on different compaction levels of chromatin^1,2^. Deformation into chromatin compaction would affect artificially gene expression as it is in case of progeria for instance. Progeria is derived from the Greek words pro (προ) and geras (γέρας), meaning premature aging which is a specific type of progeroid syndrome called Hutchinson–Gilford syndrome, a genetic disease that causes children to age rapidly within the first two years of life^3^. This happens by a mutation in the lamin-A (LMNA) gene that is responsible to make a defective protein that holds the nucleus of the cell together and causes the nuclei to be unstable which leads to multiple morphological anomalies of cell nuclei and disturbances in heterochromatin organization, mitosis, DNA replication and repair, and gene transcription. As there is no known cure, people with progeria due to complications of severe atherosclerosis, either cardiac disease or stroke, typically live to an age of mid-teens to early twenties^4–6^. It has been an interesting bio sample for image microscopy as a suitable study case to show how a deformation in the cell structures induced by a bad transcription of the genes. In fact, the most common and quantitative method available for studying this pathology is fluorescence and super-resolution techniques. To investigate for instance chromatin at nanoscale and single molecule localization^7^. However, fluorescence-based techniques are limited due to the possibility of damaging the organization of biomaterial, potential of photobleaching in high illuminating doses of light, operator dependency and requirement of expansion instruments^8^. A way to overcome such issues has been demonstrated by polarization-based microscopy techniques commonly used to enhance contrasts discerning polarimetric characteristics of anisotropic structures and chiral organization of macromolecules^9^. The technique based on employing Mueller–Stokes polarimetry formalism that confers a complete description of the optical properties of sample^10–12^. The polarimetry method linked to dichroism and birefringence of the specimen^13,14^. One of the technologies to acquire at the pixel dwell time rate in a few microseconds could be provided by the use of photoeleastic modulators^15^. The chromatin compartments and identification is in our interest since it is composed of an aggregation of proteins and chiral molecules that exhibit a strong interaction with the circularly polarized light. Understanding such molecule is crucial as it encodes all the genetic information and participates to define the organism properties. Consequently, chromatin compaction level is fascinating to investigate through polarization that is sensitive to the molecular organization, cellular/nuclear shape and very interesting if we want to diagnose genetic pathology such as progeria syndrome as a progressive genetic disorder. Nevertheless, the label free polarization technique suffers from information spatial averaging that is insensitive to different mixture of structures in the confined illumination volume defined by Point Spread Function (PSF). The main consequence is struggling to track the origin specifically the polarimetric signature of organelles at the subcellular level. Thus in case of cells label-free imaging, it becomes arduous to identify different cell types or/and identify cell structures like the chromatin in nuclear membrane. To address this problem, we denote that any modulated signal sequence can be analyzed in frequency domain by Fourier Transform mapped into a single point on a phasor plot. In early works, the phasor map introduced for super resolution microscopy as fluorescence lifetime imaging (FLIM) signal analysis and then the phasor approach extended to spectral imaging^16–18^. In the meantime, only few works have reported the use of phasor plot for label-free microscopy imaging techniques as for Second Harmonic Generation (SHG) microscopy^19^. In our previous work, we have proposed for the first time a simple theoretical study based on a phasor data analysis that exhibits remarkable capability for recognizing different structures that exhibit a strong birefringent and dichroic response in turbid medium. In parallel, we have propose to apply this approach by measuring the polarimetric fingerprint of isolated cell nuclei in a single point spectroscopic architecture by acquiring the angular scattering modulated signal using a rotational detection arm^20^. Here, we proposed to adapt our previous approach into an imaging architecture and we show that it provides on a robust and distinctive analytical approach sensitive to the polarimetric variations of different type of particle located into the PSF volume. Here, the image acquisition is coupled with a two-photon-excited fluorescence (TPEF) modality as a benchmark technique of the presence of genetic material through the PSF volume. It is worth noting that the data represented on the phasor analysis is only coming from the interaction between the polarized light and the chiral structures. In our work, the fluorescence images are presented as a benchmark of the lamin-A and chromatin organization and are compared with the polarization-resolved images. We validate primarily the analytical approach by imaging starch granules as deterministic optical active complex bio-particles excellent for highlighting the sensitivity of our approach with segmentation of the different microscopic molecular regions as phasor guideline map. Then, we present employment of the image phasor approach of polarimetric images to follow different stages of isolated Hutchinson–Gilford progeria syndrome. In an intuitive and direct way, the phenomenological polarization response changes induced by deformation of the nucleus in presence of the pathology compared to normal cells. The supremacy of image phasor map over the previous reported single point one is to deal with points indicating a finest contrast mapped from the polarimetric microscopy images in sub-microscopic level. In this paper, we illustrate sturdy polarization response of such syndrome with respect to the control normal cell compared to the confocal fluorescence images together and microspheres as reference alongside the related statistics and interpretations. Overall, we propose a graphical highly sensitive analytical method for polarimetric images in order to recognize the discrimination at different stages of development in cells.

## Materials and methods

### Multimodal polarization-resolved and fluorescence optical scanning microscopy setup

**Figure 1:**
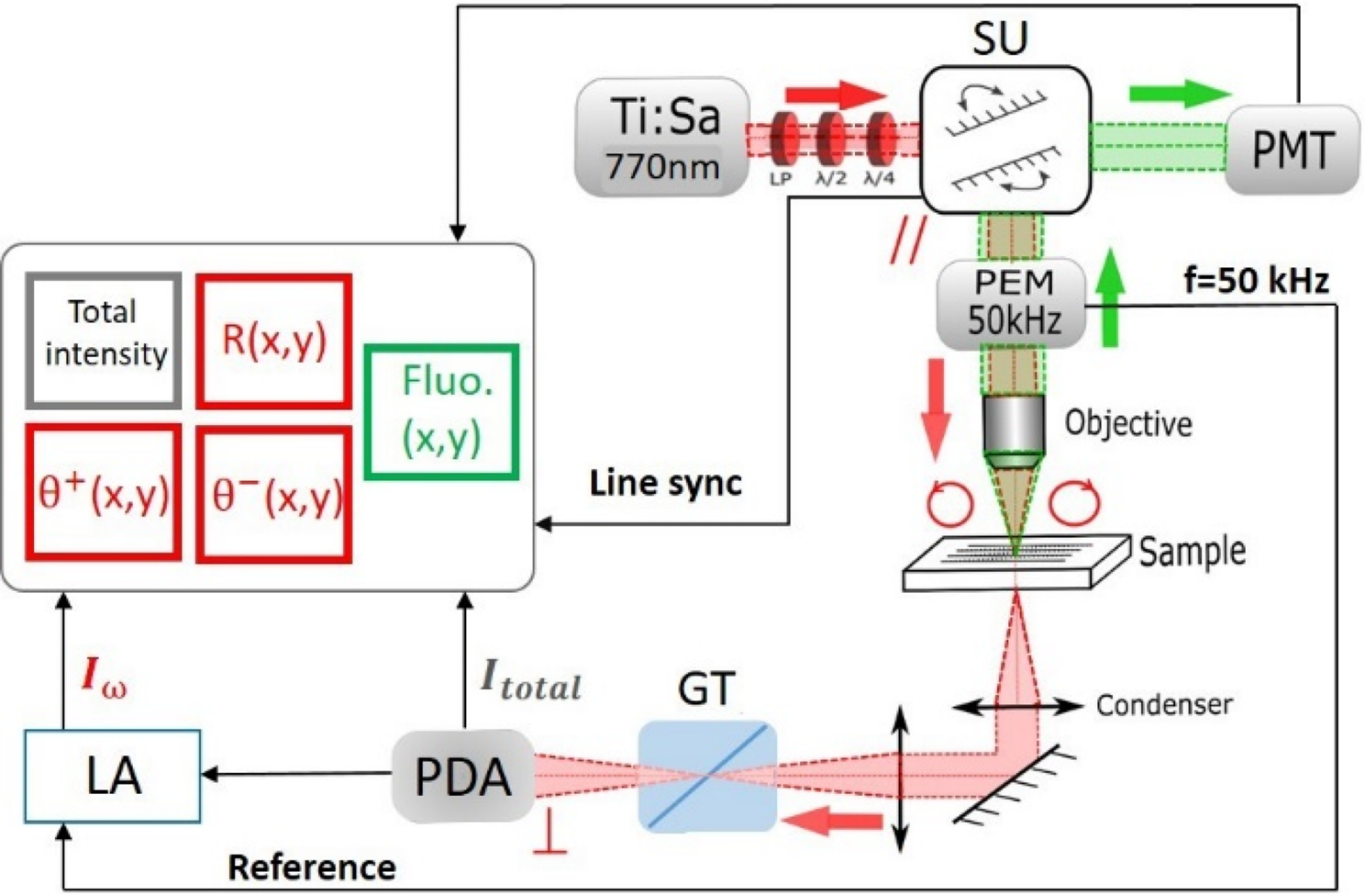
Block diagram of the bimodal scanning microscopy setup; Ti:Sa: Titanium–Sapphire coherent laser source tuned at 770 nm. *SU* scanning unit, *PEM* photoelastic modulator at 50 kHz resonant frequency, *GT* Glan–Taylor prism, *PDA* photodiode, *LA* lock-in amplifier, *PMT* photon multiplier tube. The red and green colors indicate optical path correspond to the transmitted polarimetric and to the reflected fluorescence path, respectively. ${\theta }^{+}$, ${\theta }^{-}$ representing positive and negative parts of phase of the demodulated signal.

The multimodal microscope used for the experiments schematized in Fig. 1.

The setup is composed of a polarization resolved and confocal Two-Photon-Excited Fluorescence (TPEF) imaging microscopy modalities. We illuminate sample in near infrared region at λ = 770 nm for measuring the polarized signal from biological samples representing an optical activity by a Ti:Sa femtosecond pulsed laser (Chameleon Ultra II Coherent Inc., Santa Clara, CA, USA) with a beam size of 1 mm. The illuminating wavelength is suitable to be absorbed by fluorescent Hoechst 33342 nucleic acid stain that emits blue fluorescence through two-photon excitation process when bound to the interested material. A modified Nikon scanning microscope with a fast polarization state generator (PSG) based on a Photoelastic Modulator (PEM) used to modulate the polarization of the light at a resonant frequency of 50 kHz added up along with a customized transmission detection module that allows extracting the polarized contrast pixel by pixel. PSG is composed of a linear polarizer (ColorPol® VIS 600 BC5 CW01, contrast > 100,000:1, Codixx AG, BRD), half-wave plate (HWP, PO-TWP-L2-25-UVIR, Thorlabs, Inc., USA) and quarter-wave plate (QWP, PO-TWP-L4-25-UVIR, Thorlabs, Inc., USA) in order to compensate the scanning unit (SU) optical features signature. In this way, the polarization at the PEM plane is made up by a linearly polarized light and the PEM is oriented at 45° (PEM-100, Hinds Instruments Inc., Hillsbro, OR, USA), resulting a time varying intensity signal. We used a 20X/0.5NA Nikon objective (DIC-M Plan Fluor, Nikon Instruments, Yokohama, JP) to focus the light on the sample placed in the focal plane with a diffraction-limited lateral resolution of ≈ 1.22 µm. The light then collected by a 4X/0.13NA Nikon objective (CFI Plan Fluor, Nikon Instruments, Yokohama, JP) as a condenser. After interaction with the sample, the transmitted light collected then at the crossed polarization orientation with respect to the initial LP by a tunable gain photodiode array PDA (PDA36A-EC Thorlabs, Inc., USA). The modulated signal acquired by three-channel Nikon control unit (C2+, Nikon Instruments, Yokohama, JP). Two channels synchronously coupled to the amplitude, phase of the demodulated signals at the first harmonic of the PEM applying Lock-in Amplifier (HF2LI Zurich Instruments AG, SUI) pixel-by-pixel to provide an image phasor map graphical analysis in a post-processing imaging step via specialized homemade routine in Matlab program (Matlab, v.R2017a, Mathworks). One channel connected to a conventional confocal fluorescence module collected the TPEF emission in the back reflection direction. A calibration method has been implemented and described in our previous work to take into account the optical features, any misalignment of the optical orientations of the devices of the microscope, fingerprint of the setup and to make sure that the objective does not affect the polarization as reported^21^.

### Calibration steps

**Figure 2:**
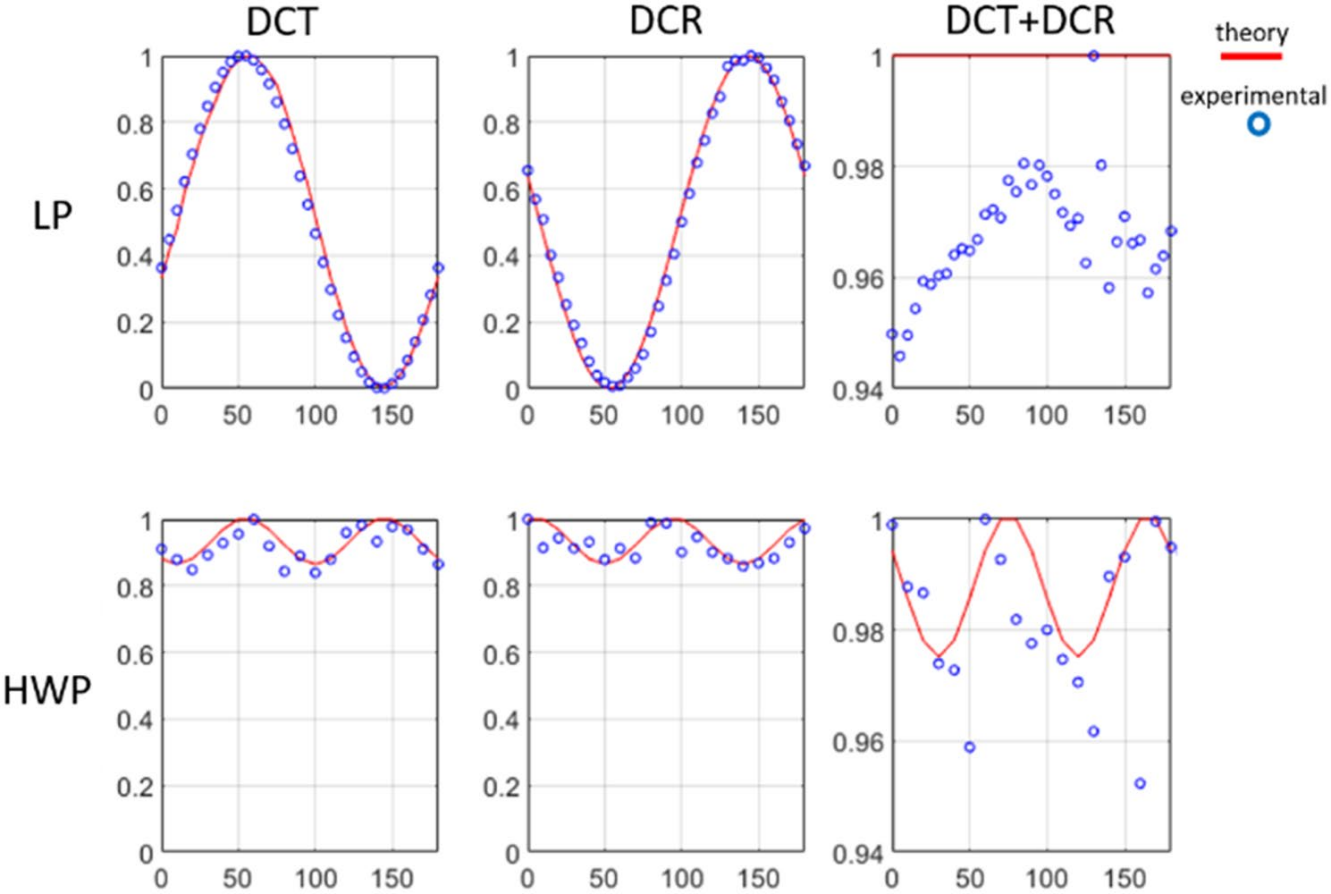
Calibration steps using LP and HWP drawn for detected normalized intensity signal versus azimuthal angle compared to the related theoretical expected curves. The red line and the points plots correspond to the theoretical and experimental data, respectively.

We have implemented a calibration step to take into account any possible remaining optical orientation misalignment of the PSG and PSA in the optical setup. Affections related to scanning configuration leading to loss of generated circular polarization in field of view (FOV) explained by Fresnel law or objective perturbations as systematic errors and the reflection at the interface of glass with sample^22^. We compensate any illumination gradient of FOV by zooming 10 times in the center. Total intensity light measured by two photodiodes (PDA36A—Si Switchable Gain Detector, Thorlabs, Inc., USA) after light splits into two orthogonally polarized beams (with respect to initial LP in PSG) by a Glan-Taylor (GT10—Thorlabs, Inc., USA) prism. We rotated LP and HWP from 0° up to 180° with the steps of 10° and measured the two intensities provided by the GT prism as ${I_\bot }$ (DCT) along with ${I_{||}}$ (DCR) and combination of them (DCT + DCR) as total intensity signal at each azimuthal angle. Finally, the intensities are averages overall the FOV and the results is reported Fig. 2.

The results in Table 1 indicate corresponding error percantages introduce by the optical features fingerprint below 10% for each channel and the sum of both channels from expected values. This guarantee that for DCT channel intensity aqcuired in this work we stay below the maximum acceptable error.

**Table 1:** Maximum polarimetric error percentage of the LP and HWP from the corresponding expected value.

	DCT error (%)	DCR error (%)	DCT + DCR error (%)
LP	6.1	4.3	5.4
HWP	7.8	9.8	4.7

### Sample preparation

Microspheres of 15 μm in polystyrene (SIGMA Chem. Corp., St. Louis, MO) are used to calibrate the instrument polarimetric properties. The refractive index of the spheres at the excitation wavelength (770 nm) is 1.58. The microspheres were suspended in dilute solution of deionized water (concentration 1:10,000). The starch granules (SIGMA Chem. Corp., St. Louis, MO) are used as reference chiral sample. The starch granules were suspended in dilute solution of deionized water (concentration 1:1000) solution at room temperature (20 °C). Then, a drop of a dilute starch–water suspension was placed on a microscope slice and fixed by a coverslip.

Human embryonic kidney 293 cells (HEK 293) were used to obtain progeria cellular phenotype. The control cell line was wild type HEK 293. Cells were grown in complete medium Dulbecco’s Modified Eagles Medium (DMEM High-Glucose, Gibco) + 1% l-Glutamine, supplemented with 10% fetal bovin serum (Sigma-Aldrich), 1% non essential aminoacids and 1% pen/strep, with 5% CO_2_, 90% relative humidity at 37 °C. To obtain the progeria cellular phenotype HEK 293 cells were stably transfected with the plasmid simultaneously encoding for the mutant protein Δ50 lamin-A and enhanced green fluorescent protein (eGFP) (Addgene plasmid #17653). Wild type HEK293 and progeria cells were plated at 50% confluency on 18 mm coverglass, previously threated with a solution of poly-l-lysine (0.01%, Sigma-Aldrich) for 10 min to promote adhesion, and grown overnight. After fixation with 4% PFA for 10 min at room temperature cells were rinsed twice for 5 min with fresh PBS. Then, cells were stained with a solution 4 μM of Hoechst 33342 (10 mg/mL, ThermoFisher) for 20 min and rinsed twice with PBS. Next, wild type lamin A in HEK293 cells was stained using conventional immunostaining protocol. After fixation, cells were incubated in Blocking Buffer (3% Bovine Serum Albumin, 0,2% Triton 100X in PBS) for 1 h at RT. The incubation with primary antibody (anti-lamin A antibody, ab26300 from AbCam, dilution 1:1000, in 3% BSA in PBS) was performed overnight at 4 °C. After three washes in PBS, the sample was incubated with secondary antibody (AlexaFluor 488, dilution 1:200, in 3% BSA in PBS) for 1 h at RT. Next, cells were washed three times with PBS. Samples were mounted on glass slides using 10 μl of Prolong Antifade mounting medium and then were ready to image.

### Data analysis and statistics

In this work, the polarization-resolved microscopy technique is categorized as temporal domain encoding polarization states and decodes the transformed ones by using the Stokes–Mueller formalism (“Multimodal polarization-resolved and fluorescence optical scanning microscopy setup”) to extract the polarization fingerprint of specimen^10^. The outcome signal is the result of the interaction between the circular polarized light and the sample thus, the resulting intensity can be described by

1 $$I\left(t\right)= {I}_{DC}+{I}_{\omega }.\mathrm{cos}\left(\omega t\right)$$

where ${I}_{DC}$ is the DC intensity of the modulated signal and ${I}_{\omega }$ is the first harmonics of AC modulation intensity. The signal then is demodulated through a lock-in detection, originating the outputs of the mixers pass through configurable low-pass filters, resulting in the two outputs real and imaginary part components ${I}_{\omega }^{Re}$, ${I}_{\omega }^{Im}$ called the in-phase and quadrature component, respectively. Using a lock-in-amplifier linked to the Nikon C2 + controller, the modulation (R) and phase (${\theta }^{\pm }$) images are obtained simultaneously in 3 channels according to Eqs. (1–5).

2 $${I}_{\omega }\equiv R=\sqrt{{g}^{2}+{s}^{2}}$$

3 $$\theta \equiv \pm \mathrm{arctan}\left(\frac{s}{g}\right)$$

4 $$g=R.\mathrm{cos}\left(\theta \right)$$

5 $$s= R.\mathrm{sin}\left(\theta \right)$$

The images reassign pixel-by-pixel through NIS Elements software. In this way, the demodulated signal is describing through the (g, s) phasor representation where we note that ${I}_{\omega }$ is equivalent to modulation $R$ derived from g and s. The phase $\theta$ acquired by a transformation from cartesian coordinates into polar coordinates described by Eq. (3) in order to have an output range for the phase angle that covers all the angles, from − π to π. Therefore, it is dealing with circular polarization response of the matter but the result differs from Circular Intensity Differential Scattering (CIDS) that parallel polarized light at output also taken into account^21^. It worth noting that the temporal input signal channel split separately and multiplied with reference signal of PEM modulated at frequency f = 50 kHz and 90° phase-shifted copy of it. Since NIS element software does not provide negative values so the phase is shifted the second channel to take the absolute data to be post-processed by subtracting the first channel from the second one. The results of polarization-resolved microscopy is interpreted using MM formalism. To this target, we resolved polarization characteristic of the sample related to its linear birefringence and circular dichroism signature corresponding to the detected signal^21,23^. We validated the interpretation of the results by statistics of samples composed of normalizing the list of intensities by the maximum value and calculating mean of them to present along with standard deviation (STD) in all the pixels of images at a Region Of Interest (ROI) for both $R$ and $\theta$.

#### Image processing

The retrieved modulation $R$ simply normalized, ${R}_{N}(x,y)$ for each pixel by the maximum value and positive/negative phase (${\theta }^{\pm }$) then normalized ${\theta }_{N}$ by the STD multiplied with the maximum of $\theta$ as follows:

6 $${R}_{N}(x,y)=\frac{R (x,y)}{{R}_{max}(x,y)}$$

7 $${\theta }_{N}=\frac{\theta }{std*\mathrm{max}(\theta )}$$

We determine manually the background by an arbitrary polygonal over the image from a Matlab routine. The intensities of the whole pixels of the image subtracted from the average intensity of pixels corresponding to the background to remove the gradient due to the inhomogeneous intensity FOV for both normalized phase and modulation in order to present as phase/module 2D images and related phasor analysis. We determine also thresholds of modulation and phase for data segmentation depicted by different colors in ROI.

## Results and discussions

### Image phasor analysis

The phasor of polarized modulated signal $I\left(t\right)$ pixel-by-pixel provides a 2D graphical view of the demodulated signal at reference frequency in terms of amplitude and phase distributions. The phasor coordinates (g, s) related to the product of intensity of the demodulated first harmonics via Fourier transform of the signal by sine and cosine terms for each pixel, as the modulation (R) and phase (θ) values, by the trigonometric relations (Eqs. 3, 4).

In our case, the modulation and phase for each pixel supplied via Lock-in Amplifier (LA) by the convolution of sine and cosine terms as remarked in previous section. Sampling then settled corresponding to the pixel-dwell time (20 μs) of signal detector pixel-by-pixel. According to the definition, the phasor associated to the modulated signal has coordinates $g\left(\omega \right), s\left(\omega \right)$ is given by

8 $$g\left(\omega \right)= \frac{I\left(t\right) \otimes \mathrm{ cos}(\omega .t)}{\mathrm{max}[I\left(t\right)]}$$

9 $$s\left(\omega \right)= \frac{I\left(t\right) \otimes \mathrm{ sin}(\omega .t)}{\mathrm{max}[I\left(t\right)]}$$

where $\omega =50 kHz$ is the modulation angular frequency of the reciprocal time dependent signal used for the Fourier Transform and each frequency dependent component X and Y normalized by maximum $I\left(t\right)$ value. The vertical axis $s\left(\omega \right)$ shows imaginary and the horizontal one $g\left(\omega \right)$ indicates real parts of the first harmonics. The phasor approach defined R between 0 and 1, and φ between 0 and 2π.

### Validation on reference samples

#### Polarized-resolved phasor analysis of starch granule

Any specimen whose refractive index depends on polarization propagation direction of light is said to be birefringent that shows a characteristic variation of intensity of light after passing through a polarizer. Starch granules are products of living particles that has peculiar structures called amylopectin, responsible for a special optical active response of the particle based on their angle of orientations in terms of the anisotropy, degree of polarization and depolarization effects. They have been studied with advanced microscopy techniques for revealing molecular structure, such as in scanning electron microscopy (SEM), second harmonic generation (SHG) microscopy and atomic force microscopy (AFM)^24–26^. It has been shown since starch granules exhibit positive birefringence and crystallinity is aligned in radial directions, the molecular symmetry of starch granules inferred from the output polarization directly.

**Figure 3:**
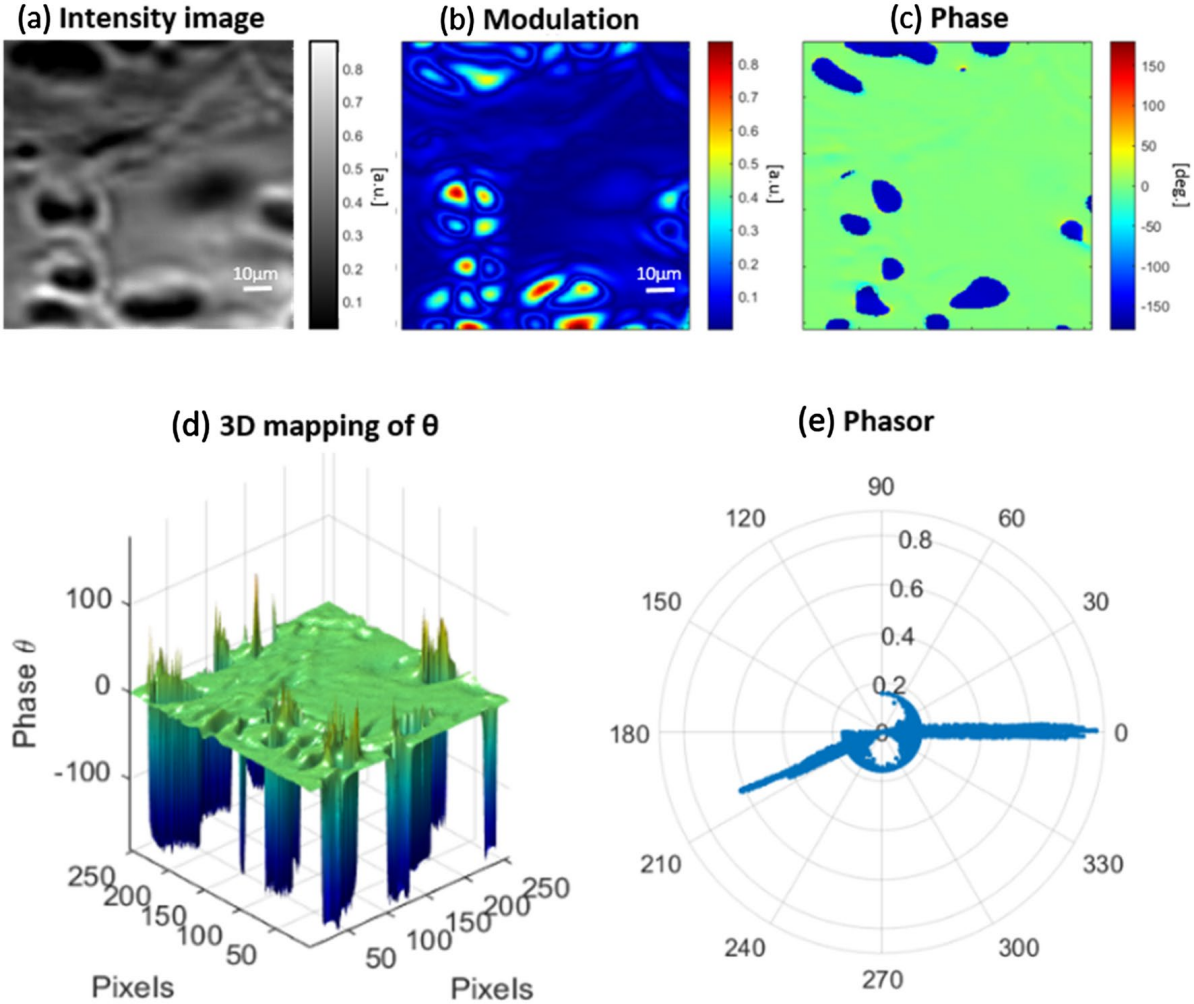
(**a**) Total intensity (absorption) image of starch granules compared to (**b**) corresponding module polarization-resolved one. (**c**) 2D phase microscopy images of starch granules. The color scale shows the values of parameters. (**d**) 3D phase microscopy image. (**e**) Corresponding image phasor map.

Thanks to the starch granules optical structure, the abundance of different optical active regions confer to this specimen interesting properties for our phasor approach. Indeed, it provides imaging polarimetric contrast overall the FOV and segmentation 2D imaging as case study sample for indicating the phase and modulation characteristics. We examine our microscopy method and analysis approach by such known starch granules based on sensitivity and recognition of molecular formations in different microscopic regions pixel-by-pixel (Fig. 3).

**Figure 4:**
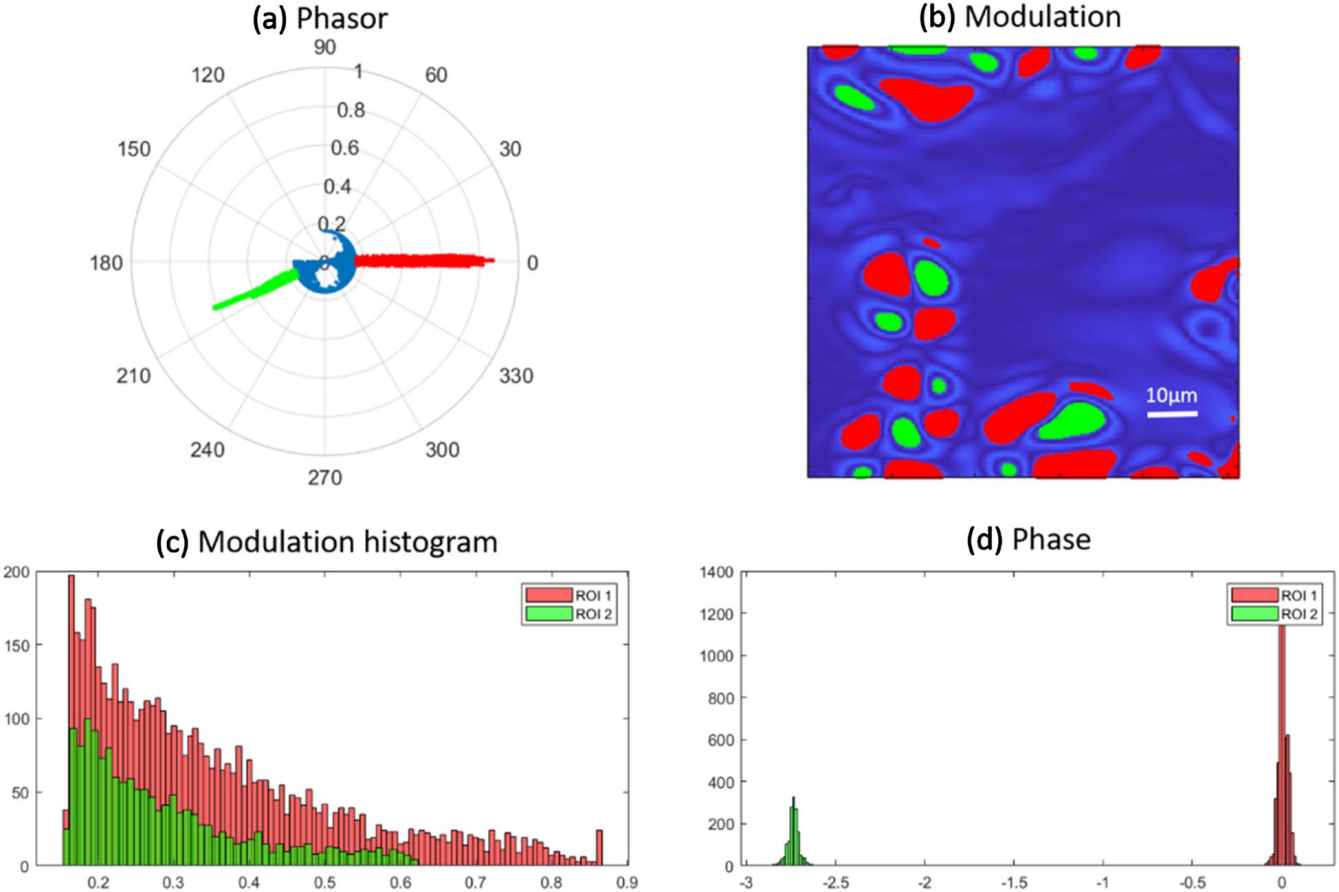
(**a**) Segmentation of different regions in phasor map graph and (**b**) corresponding 2D modulation image. (**c**,**d**) Histogram of different regions of interest marked by green and red colors for modulation and phase effects.

The modulation R image exhibits cluster of the granules in order to increase the modulation effect to shown in the polar phasor map. The 3D mapping of the 2D phase image emphasizes the difference between above-mentioned areas. The phasor yielded as cluster of points each mapped from a pixel of polarization resolved image in terms of normalized modulation amplitude and phase. According to the image phasor map, we observe elongated modulations at 0° and around 210° intersected from the center of the phasor by a cluster of circle shaped at certain threshold as radius less than 0.2. In Fig. 4a–c, we proposed to use the phasor approach as a tool for tracking a specific region of the sample from the segmentation of the phasor plot. It worth noting that the variations in shape and size of the starch observed in Fig. 3b are linked to different condition of degradation of the sample due to external factors like temperature fluctuations.

The red and green parts correlate with quadrature birefringent regions in starch and the blue one is representing background obviously. This proves that we have minimum modulation effect less than the radius of the blue circle like phasor and maximum inside the crystalline granule structures. We also calculated the abundance histogram of the image in terms of modulation and phase that specifies the dominant role for the modulation at 0° with respect to the fellow one at around 210°. In previous literatures, the chirality for starch crystals is still not determined clearly and we observe that in this work, they seems to exhibit a preferentially left circular polarization (LCP) emission. Accordingly, only zero and negative phase values are observed from the starch crystals. We concluded that the inside four separate regions on the granules there is only active left circular polarization effect shown in Figs. 3b,c and 4d.

#### Polarized-resolved phasor analysis of progeria vs normal cell nuclei

It was reported that the polarization resolved signal is sensitive to a long-range organization of chiral structures like chromatin^27^. Therefore, in order to investigate chromatin compaction as highly chiral material inside a normal isolated HEK 293 cell nucleus, circular polarization resolved microscopy method has been integrated in a homemade confocal fluorescent microscope similar to the one reported in our previous works^21,23^. Here we layout a new paradigm to use image phasor map analysis as a robust and effortless tool to distinguish the progeria from normal HEK cell nuclei based on difference in chromatin compaction, spatial organization of chromatin inside the nucleus and deformation of lipid double membrane that encloses all the nuclear genetic material. Consequently, birefringence and dichroism changes can be discriminated in a simple graphical manner and pixels of the image are reported one-by-one into the reciprocal frequency space.

**Figure 5:**
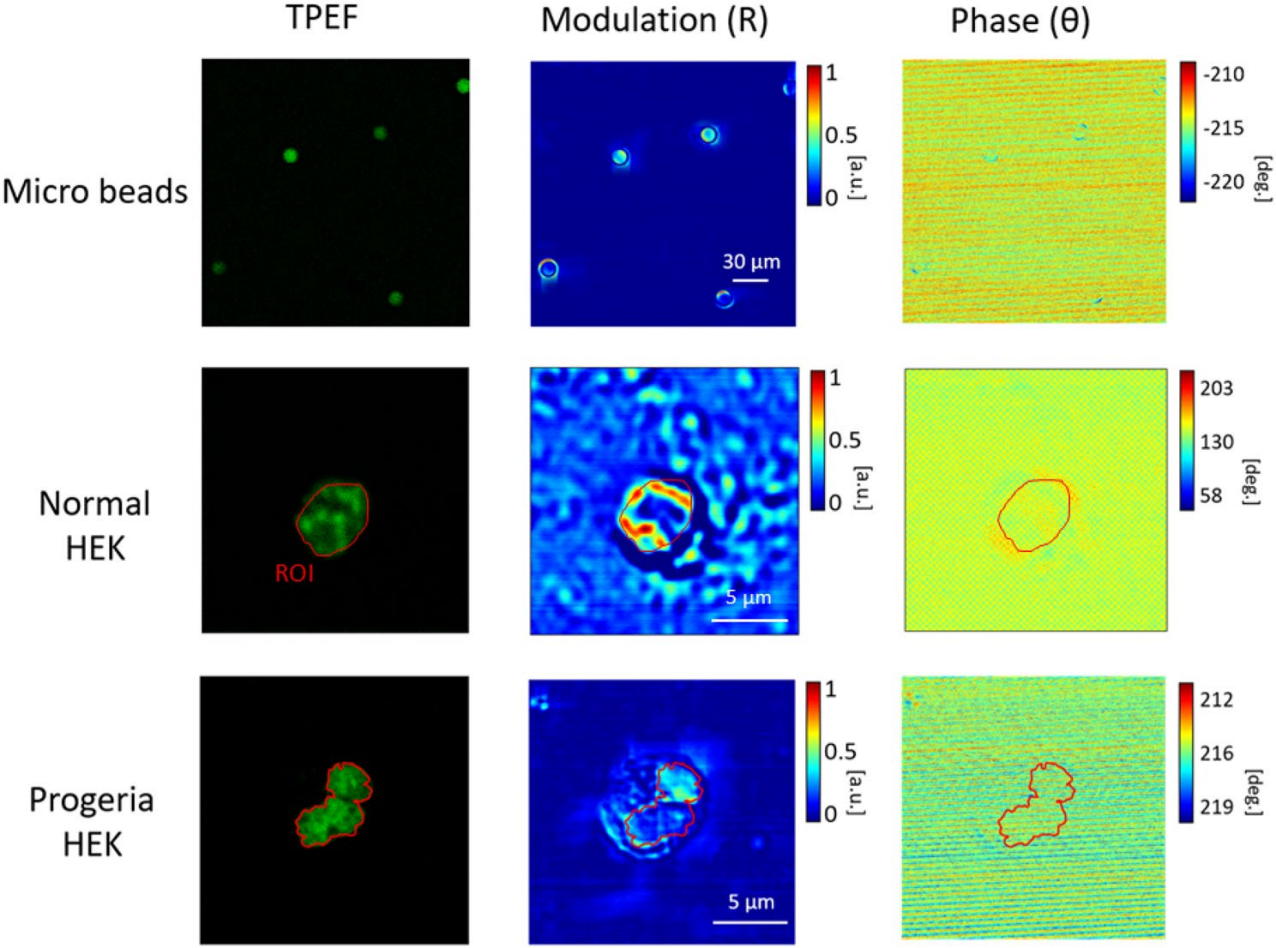
Fluorescent images of microbeads as reference, the isolated normal HEK nucleus and progeria HEK syndrome compared to corresponding polarimetric modulation and phase images.

The concluding aim is to observe directly the discrimination of normal cells with progeria images pixel-by-pixel exploiting our image phasor map approach. The samples labelled by Hoechst indicates presence of DNA-based molecules only inside the nuclear membrane. Figure 5 exhibits the confocal two-photon excitation fluorescent (TPEF) and polarization-resolved microscopy images of microspheres as reference, HEK cell nuclei and progeria respectively. We exploit the fluorescent images to identify the ROI and report to modulation and phase microscopy ones. We provide also the results for fluorescent 15 μm beads as a baseline reference in the polarization measurements and corresponding modulation with phase images.

**Figure 6:**
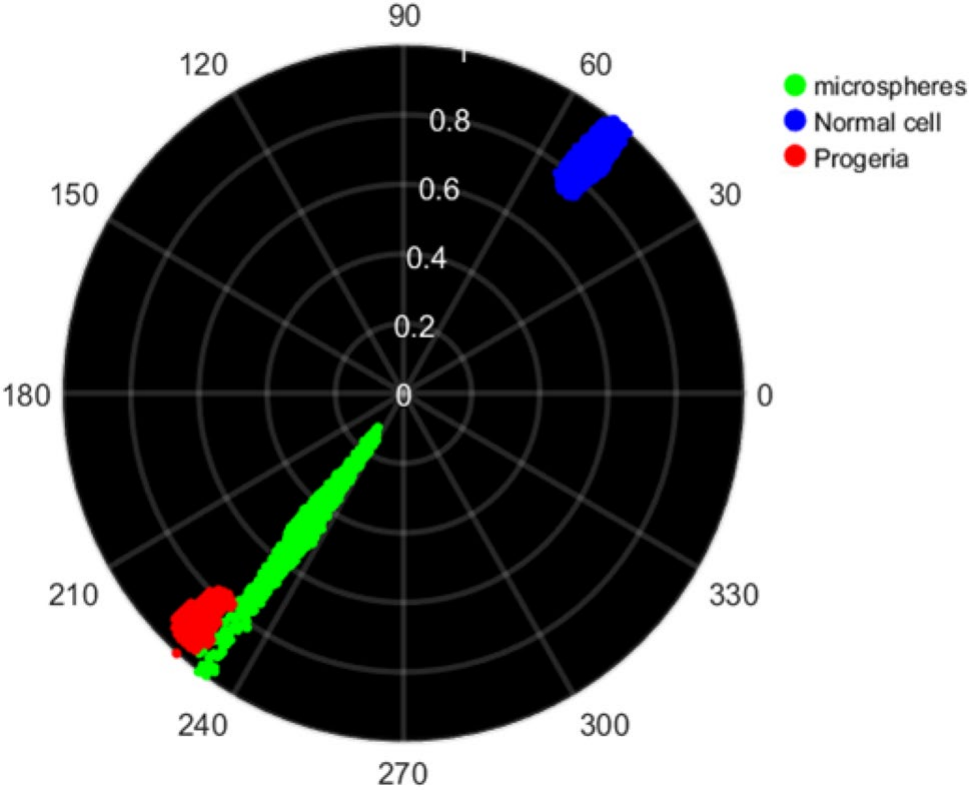
Image phasor map of microspheres as reference, control normal HEK cell data compared to progeria HEK cell syndrome discriminated by green, blue and red respectively.

In consequence, we show that modulation in progeria HEK cell is weaker than the normal one. The situation goes even weaker when dealing with phase images so that the phase image of the progeria almost vanishes according to trivial optical phase changes inside the nuclei. This is the main point that makes it possible to discriminate normal HEK from the progeria one on the phasor map representation. We discern very low contrast in terms of modulation and phase images since the less compacted chromatin is inside the deformed shape of the nuclear membrane so a stronger scattered light is produced by the deformed shape of the entire cell. The values of each pixel in modulation and phase images then normalized to the map on image phasor point-by-point shown in Fig. 6.

**Figure 7:**
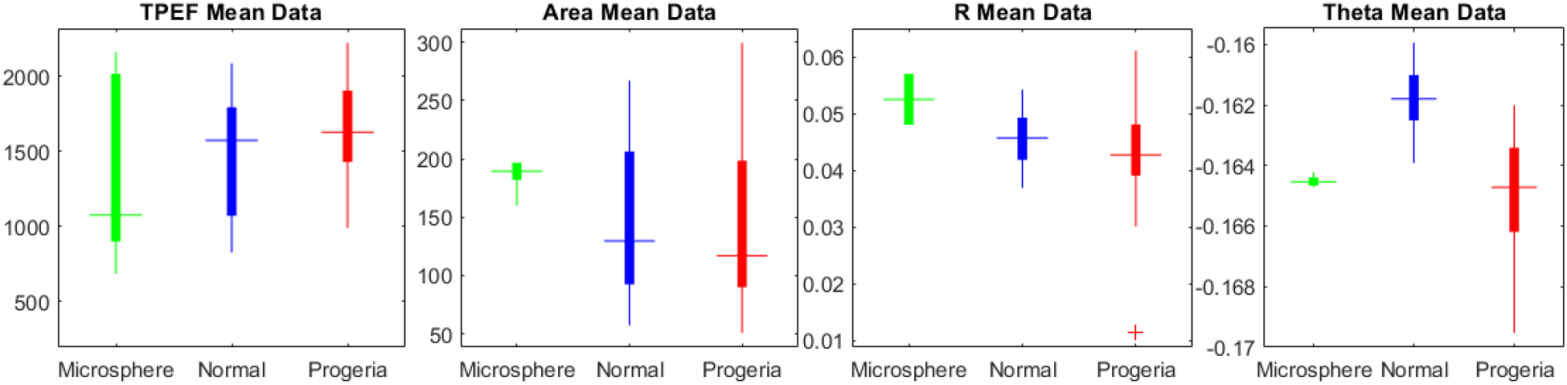
The statistical analysis to discrimination to indicate the sensitivity of the phasor approach. The size of the nucleus, TPEF intensities, modulation, phase mean values, then corresponding skewness and kurtosis. There is more sensitivity for phase rather than modulation as the phase is link to the retardance.

**Figure 8:**
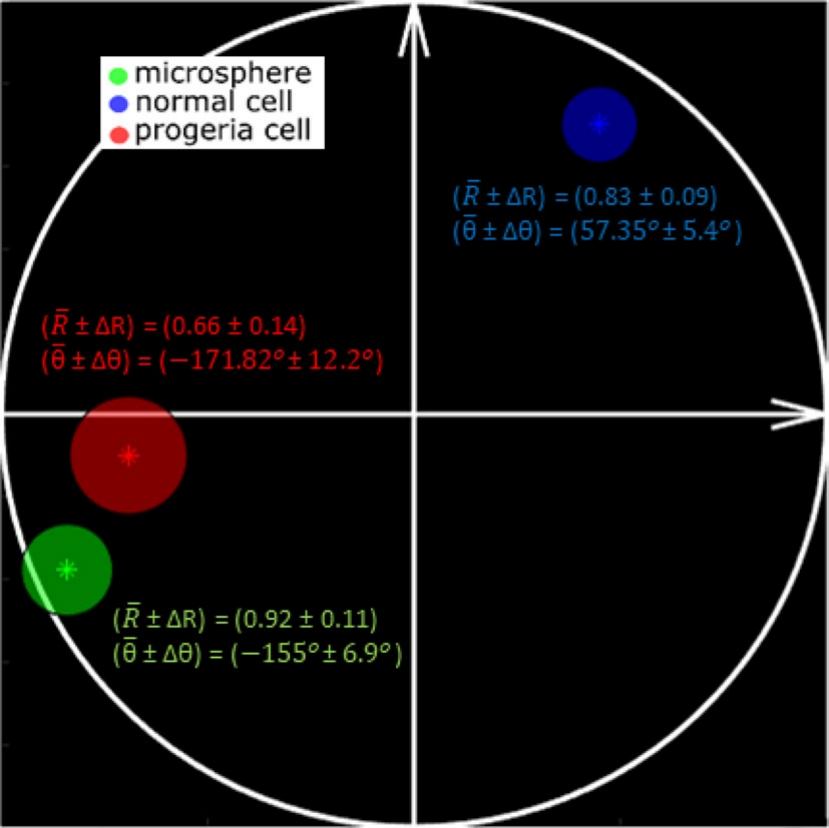
Image phasor map analysis on the statistical information out of microspheres as reference, normal HEK cell and progeria HEK syndrome determined by green, blue and red circles. The center of each represents mean value and the radius shows the std.

Accordingly, image phasor map is capable of discerning the differences based on chromatin condensation and morphology deformation of nuclear membrane of the progeria and normal one in terms of phase and modulation changes after normalization by the maximum values. The modulation R of the reference microspheres phasor elongated from almost 0.2 that is related to the threshold of background region determined in Fig. 5a, up to 1 and the phase θ is non zero since for such perfect isotropic spherical particles, where illuminating edge effects dominates the modulation and phase variations. It worth noting that the phasor is only dependent on polarimetric signal since application of different fluorescent types dose not affect the image phasor and any variations based on phasor map is trivial compared to our scale. We notice the discrimination between them in a glance that proves the approach as a robust and applicable gadget as proved in Figs. 7 and 8.

We observe the size distribution more dispersed for the progeria rather than the normal cells that means the progeria shapes present more diversity as we expected. In case of TPEF, the distribution is more different for the normal nuclei from the progeria one since the asymmetric 3D shape of the progeria ends up losing the fluorescence signal so that makes it more difficult to have the entire fluorescence in the whole cell compare to the normal cells. The results show that since the progeria has a 3D distribution more random than the normal nuclei, it could lead to reduce the quality of the circular polarization in the illumination PSF volume. Therefore, it is suggested to investigate only the mean value that presents a superior discrimination between the normal cells and progeria ones. The corresponding phasor plot in Fig. 8 averaged over a statically significant population of 30 isolated progeria images compared to the same number of the normal cell nuclei. The resultant analysis for microspheres, the normal HEK cells nuclei and the progeria depicted graphically applying the image phasor approach for normalized modulation R and phase. The radius of colored phasors indicate the STD for each. Consequently, we are able to separate the phasor of the normal cell and progeria one in the image phasor based on mean valued quantities. It shows clearly that the dispersion is higher in case of the progeria rather than for the control normal samples and then the microsphere as reference.

## Conclusion

The image phasor map approach presents an easy, robust and intuitive graphical analysis to discriminate different regime of highly compacted bio-macromolecules using polarization-resolved optical scanning microscopy. In this work, we integrated a polarimetric imaging modality into a confocal fluorescent optical microscope and analyzed data by statistical methods for several trials to interpret and graphically shown by our image phasor map in terms of birefringence and dichroism. We initially apply the polarimetric imaging method on starch granules that are complex crystals, analyze them by segmentation of disparate optical active regions and represent graphically employing the corresponding image phasor map as baseline intuitive for any general imaging. The requisition of the introduced phasor approach on chiral biosamples made us able to discriminate normal HEK cells from progeria syndrome according to chromatin compaction and nuclei morphology deformations for the first time using a multimodal optical scanning microscopy. This work would pave the way to make extremely sensitive user-friendly mechanism to track microscopic objects down to tens of nanometers in real-time such as either different microscopic species or phenotypes of a certain virus in the next coming future.

## References

[1] Gilbert N, Boyle Sh, Fiegler H, Woodfine K, Carter N, Bickmore W (2004). Chromatin architecture of the human genome: gene-rich domains are enriched in open chromatin fibers. Cell.

[2] Huisinga K, Toland B, Elgin S (2006). The contradictory definitions of heterochromatin: transcription and silencing. Chromosoma.

[3] Roach, E. S., & Miller, V. S. Neurocutaneous disorders. Cambridge University Press (2004).

[4] Ashapkin V, Kutueva L, Kurchashova S, Kireev I (2019). Are there common mechanisms between the Hutchinson–Gilford progeria syndrome and natural aging?. Front. Genet..

[5] Hsiao, K. Advances in clinical chemistry. Academic Press, vol 33, p. 10 (1998).

[6] Eriksson M (2003). Recurrent de novo point mutations in lamin A cause Hutchinson–Gilford progeria syndrome. Nature.

[7] Flores C (2011). DNA and chromatin imaging with super-resolution fluorescence microscopy based on single-molecule localization. Biopolymers.

[8] Cornea, A., & Conn, P. M. Fluorescence microscopy: Super-resolution and other novel techniques. Academic Press.

[9] Ippolito S (2008). Polarized high-resolution imaging. Nat. Photonics.

[10] Mueller H (1948). The foundations of optics. J. Opt. Soc. Am..

[11] Snik F, Craven-Jones J, Escuti M, Fineschi S, Harrington D, De Martino A, Mawet M, Riedi J, Tyo J (2014). Measurement of the optical activity of anisotropic samples by transmission Mueller matrix ellipsometry. Polariz. Meas. Anal. Remote Sens..

[12] Le Gratiet A, Mohebi A, Callegari F, Bianchini P, Diaspro A (2021). Review on complete mueller matrix optical scanning microscopy imaging. Appl. Sci..

[13] Le Gratiet A, D’Amora M, Duocastella M, Marongiu R, Bendandi A, Giordani S, Bianchini P, Diaspro A (2019). Zebrafish structural development in Mueller-matrix scanning microscopy. Sci. Rep..

[14] Huber E, Baltzer N, von Allmen M (1985). Polarization modulation ellipsometry: A compact and easy handling instrument. Rev. Sci. Instrum..

[15] Le Gratiet A, Marongiu R, Diaspro A (2020). Circular intensity differential scattering for label-free chromatin characterization: A review for optical microscopy. Polymers.

[16] Digman M, Caiolfa V, Zamai M, Gratton E (2007). The phasor approach to fluorescence life time imaging analysis. Biophys. J..

[17] Scipioni L, Bona M, Vicidomini G, Diaspro A, Lanzanò L (2018). Local raster image correlation spectroscopy generates high-resolution intra celular diffusion maps. Commun. Biol..

[18] Fereidouni F, Bader A, Gerritsen H (2012). Spectral phasor analysis allows rapid and reliable unmixing of fluorescence microscopy spectral images. Opt. Express.

[19] Radaelli F, D’Alfonso L, Collini M, Mingozzi F, Marongiu L, Granucci F, Zanoni I, Chirico G, Sironi L (2017). μMAPPS: A novel phasor approach to second harmonic analysis for in vitro-in vivo investigation of collagen microstructure. Sci. Rep..

[20] Mohebi A, Le Gratiet A, Marongiu R, Callegari F, Bianchini P, Diaspro A (2021). Combined approach using circular intensity differential scattering microscopy under phasor map data analysis. Appl. Opt. (OSA).

[21] Le Gratiet A, Pesce L, Oneto M, Marongiu R, Zanini G, Bianchini P, Diaspro A (2018). Circular intensity differential scattering (CIDS) scanning microscopy to image chromatin-DNA nuclear organization. OSA Contin..

[22] Le Gratiet A, Dubreuil M, Rivet S, Le Grand Y (2016). Scanning Mueller polarimetric microscopy. Opt. Lett..

[23] Marongiu R, Le Gratiet A, Pesce L, Bianchini P, Diaspro A (2020). ExCIDS: A combined approach coupling expansion microscopy (ExM) and circular intensity differential scattering (CIDS) for chromatin-DNA imaging. OSA Contin..

[24] Jane J, Kasemsuwan T, Leas S, Zobel H, Robyt J (1994). Anthology of starch granule morphology by scanning electron microscopy. Wiley.

[25] Zhuoa Z, Liao C, Huang C, Yu J (2010). Second harmonic generation imaging—A new method for unraveling molecular information of starch. J. Struct. Biol..

[26] Ridout M, Gunning A, Parker M, Wilson R, Morris V (2002). Using AFM to image the internal structure of starch granules. Carbohyd. Polym..

[27] Finzi L, Ulibarri L, Bustamante C (1991). Differential polarization imaging. V. numerical aperture effects and the contribution of preferential scattering and absorption to the circular dichroism images. Biophys. J..

